# Conception Preferences during COVID-19 Pandemic Lockdowns

**DOI:** 10.3390/bs12050144

**Published:** 2022-05-13

**Authors:** Soha Albeitawi, Zina Al-Alami, Khaldoun Khamaiseh, Lama Al Mehaisen, Almu’atasim Khamees, Jehan Hamadneh

**Affiliations:** 1 Clinical Science Department, Faculty of Medicine, Yarmouk University, Irbid 21163, Jordan; almotasem.kh@gmail.com; 2 Medical Laboratory Sciences, Faculty of Allied Medical Sciences, Al-Ahliyya Amman University, Amman 19328, Jordan; z.alalami@ammanu.edu.jo; 3 Women Health Center, Al-Ahliyya Amman University, Amman 19328, Jordan; 4 Obstetrics & Gynecology Department, Faculty of Medicine, Al Balqa’ Applied University, Amman 17705, Jordan; khamaiseh28@hotmail.com (K.K.); lmehaisen@yahoo.com (L.A.M.); 5 Obstetrics and Gynecology Department, Faculty of Medicine, Jordan University of Science & Technology, Irbid 22110, Jordan; jehan_hamadneh@yahoo.com

**Keywords:** COVID-19, coronavirus, fertility, pregnancy, assisted reproductive technique, conceiving, Jordan

## Abstract

**Background:** The COVID-19 lockdowns imposed new challenges to couples who were planning to conceive. In this research paper, we aimed to study the perceptions of women in Jordan during the pandemic regarding fertility behavior, the desire to use assisted reproductive technology (ART) and the awareness and beliefs of potential risks related to conception. **Methods:** A validated online-based questionnaire was distributed to women from April–May 2020, Statistical analysis was performed using the statistical software SPSS version 22 and R software (2020); *p* values ≤ 0.05 were considered statistically significant. **Results:** The total number of participants was 814 women, with 78.2% of the participants (58.7% fertile and 76.6% infertile) believing that pregnancy during the COVID-19 pandemic could be risky. Among them, 16% and 40%, respectively, were trying to conceive during the pandemic, and 97.4% and 89.9%, respectively, were not willing to use ART if needed during the pandemic. Young, nulliparous women who were married for less than one year were significantly associated with the desire to conceive during the COVID-19 pandemic. **Conclusion:** This study concluded that the fertility behavior of women in Jordan changed during the pandemic, and their desire for natural conception and for using ART declined, as they believed that there were potential risks related to conceiving during the pandemic. However, the effect was greater among the general fertile population than the infertile.

## 1. Introduction

Because of health, economic and social concerns, the coronavirus disease 2019 (COVID-19) pandemic has created many issues, including in family planning, people’s options, and wishes about conceiving and having children. Plentiful literature exists on how COVID-19 has affected the sexual and reproductive health of women in the world, yet not much is known about Jordan.

In a study conducted in Italy, many participants who were preparing to have a child before the pandemic changed their mind, particularly because they were worried about economic troubles and concerns regarding pregnancy [1]. In the United States of America, in a study about early effects of the pandemic on fertility preferences, about one third of the participants claimed that they changed their fertility plans [2].

In such conditions, the safety of patients, staff, and the whole society should be a priority. Therefore, the American Society of Reproductive Medicine (ASRM), the European Society for Human Reproduction and Embryology (ESHRE), the British Fertility Society, and the Italian Fertility Society have provided recommendations regarding assisted conception after the initial advice to suspend initiation of new treatment cycles and to consider cancellation of all embryo transfers at the beginning of the coronavirus pandemic [3,4]. Countries around the world imposed lockdowns in an attempt to curb this infection and flatten the rising curve of cases. As a response to the pandemic, in Australia, in March 2020, all non-critical surgeries were suspended, which had an impact on fertility treatment, since they are classified as non-critical and non-essential surgeries, and fertility clinics in the United Kingdom started cancelling assisted reproductive technology (ART) procedures [5,6].

In Jordan, a lockdown was imposed mid-March 2020. Fertility patients were confronted with a challenge due to the unexpected physical, financial, and psychological loss because of the sudden suspension of fertility treatment. Although fertility treatment seems non-essential in such circumstances, for fertility patients, it is crucial [7]. Conversely, for healthy fertile couples, lockdowns due to the pandemic gave them the opportunity to spend enough time together to have a new baby [8]. However, the fear of infection with COVID-19 and possible associated risks during pregnancy could interfere with their plans. Moreover, job loss, loss of loved ones, and deterioration in health can affect fertility decisions and plans [9]. In this research paper, we aim to study the perceptions of women in Jordan during the pandemic regarding fertility behavior, the desire of using ART, and the awareness and beliefs of potential risks related to conception.

## 2. Methods

This was an online-based cross-sectional study conducted during the lockdowns in April–May 2020 on women in Jordan who were willing to participate voluntarily in the study.

**Sample/participants**: The study was addressed to married women in Jordan. The reports published by the department of statistics in Jordan state that the total number of females in Jordan during the year 2020 was 50,840,000. The target sample size was estimated based on Raosoft software; website: http://www.raosoft.com/samplesize.html (accessed on 16th April 2022). To calculate minimal sample size needed for a 50,840,000-person population size using a confidence interval of 95%, a standard deviation of 0.5, and a margin of error of 5%, the required sample size is 385 participants. **Questionnaire validation:** After the questionnaire was prepared and drafted in the Arabic language by one of the authors (S.B.), it was reviewed by three independent infertility specialists for validation; their comments, feedback, and remarks were taken into consideration. The questionnaire was then created using the online survey platform Google Forms. Next, it was distributed to the women in Jordan via social media and by email. The questionnaire was available online starting from 11 April 2020 for four weeks. Cronbach alpha coefficient was 0.171 with a 95% CI of 0.127–0.215 for the three questions asking: if it was a risk to get pregnant during the pandemic, if the participants were trying to get pregnant during the pandemic, and if she would use ART if needed during the pandemic.

**Sections of the questionnaire**: Prior to the questionnaire’s sections, a message was delivered asking the participants to kindly and voluntarily answer the inquiries according to their knowledge and preferences at the time when they answered the questionnaire (April–May 2020). Participants who agreed to participate voluntary in the study could start the survey.

The questionnaire consisted of four sections, successively: (1) demographic data, including age, education, place of residence, occupation, years of marriage, number and gender of children, and age of the youngest child; (2) fertility behavior during the pandemic, including the desire to have more children, conception trials during the time filling the questionnaire, and if the lockdown and pandemic were good times to conceive; (3) using ART during the pandemic, inquiring about using ART previously, preferences about using ART during the pandemic, and contacting the gynecologist for their advice; (4) awareness of the risks related to conceiving during the pandemic and inquiring about the beliefs about possible health risks if a pregnant woman caught coronavirus.

**Data collection**: The questionnaire was released online via Google Forms and was spread via social media and email, starting from 11 April 2020 for a total of four weeks.

**Data Analysis**: Univariate frequency analyses and bivariate cross-tabulations were carried out for the variables of interest. The exact binomial test was used to compare the probability of selection of response options to chance probability. The Chi-square test was used to check the association between pairs of variables and the homogeneity between distributions.

Percentages of women who desire to have more children, women who are trying to have more children during the quarantine, and women who accept to use ART during the quarantine were compared with age, education level, number of current children, and number of years of marriage.

Comparisons between the desire to have more children, trying to have more children during quarantine, accepting to use ART during quarantine, and the mentioned factors were determined by using Pearson’s Chi-square test. Statistical analysis was performed using the statistical software SPSS version 22 and R software (2020), and *p* values ≤ 0.05 were considered statistically significant.

**Ethical considerations:** Only women who agreed to take part of the study answered the survey. The questionnaires were anonymous, and all data were treated with confidentiality. Ethical approval (1891/1/13) was obtained from the Institutional Review Board at Jordan University of Science and Technology, King Abdullah University Hospital, Jordan.

## 3. Results

### 3.1. Study Demographics

The total number of participants was 814 women. Table 1 shows the general characteristics of the participants. 

Out of all respondents, 619 women (76%) had children without reproductive assistance, and 138 women (17%) had used ART previously, and these groups are respectively referred to as fertile women and infertile women throughout the text. Only 57 (7%) did not have children and never sought assistance; most of them were newly married or had been married for less than four years.

### 3.2. Awareness of Risks Related to Conceiving during Pandemic

Of the 814 participants, 710 women (87.2%) answered that pregnancy could potentially pose a risk to the woman in the case of contracting the coronavirus, and 647 respondents (79.5%) did not think that the pandemic was a good time to conceive. Upon cross-tabulating these two variables, it was found that the majority of respondents (585; 71.9%) thought that pregnancy could be dangerous, could have risks, and that the pandemic was not a good time to conceive. However, although 15.4% of respondents thought that there was a potential risk to pregnancy, they still thought the pandemic was a good time to conceive. Only 5.2% of respondents thought that there were no risks and that it was a good time to conceive. Finally, 7.6% thought that there was no risk, but they did not think it was a good time to conceive. Two-way Chi-square test was conducted to check the association between awareness of pregnancy risks and whether the pandemic was a good time to conceive or not, and there was a significant association with *p* < 0.001.

Differences in the awareness of pregnancy risk were clear; a group of 138 infertile women (17%) and a group of 619 fertile women (76%) thought that getting pregnant during the pandemic may pose a risk to the woman’s health. Of the infertile women, 81 of the 138 (58.7%) and 474 of the 619 fertile women (76.6%) thought that the pandemic was not a good time to conceive and knew that pregnancy could be dangerous.

### 3.3. Fertility Behavior during Pandemic

About half of the respondents (414; 50.9%) claimed that they desired to have more children. However, 303 of the 414 women in this group (73.2%) were not trying to conceive during the pandemic. Eight women said that they desired to have more children, but they did not answer whether they were trying to conceive or not. These eight women were excluded from further analysis. This left 406 women who wanted more children and who answered whether or not they were trying to get pregnant during the pandemic. Comparing the fertile and infertile women who wanted more children, only 16% of the fertile women and 40% of the infertile women were trying to conceive during the pandemic.

### 3.4. Undergoing ART If Needed during the Pandemic

The majority of all respondents (779; 95.7%) said that they would not use ART if needed during the pandemic. The risk of pregnancy during the pandemic was given as one reason by 54.6% of this subgroup. Moreover, 20.8% of all respondents chose that the injections and operation were dangerous and might cause infections. Interruption of services during such public health emergencies might play a role as well. Using the exact binomial test, the frequencies of selection of the different options were found to be significantly different (*p* < 0.001). Comparing the fertile and infertile women, a significantly higher percentage of fertile women (603 out of 619; 97.4%) said that they were not willing to use ART if needed during the pandemic, while 124 of the 138 infertile (89.9%) were not willing.

### 3.5. Factors Related to Fertility Behavior during the Pandemic

A remarkable strong association was demonstrated among age, number of years of marriage, and parity, with the tendency to get pregnant during the COVID-19 pandemic.

There was a significant association between being less than 20 and trying to conceive (Chi-squared test: X^2^ = 22.6, df = 1, *p* value < 0.001). There was also a significant association between being nulliparous and trying to conceive (Chi-squared test: X^2^ = 72.25, df = 1, *p* value < 0.001). In addition, there was a significant association between being married less than a year and trying to conceive (Chi-squared test: X^2^ = 23.04, df = 1, *p* value = 0.0000016).

## 4. Discussion

Several factors affect fertility preferences and behavior among societies, age, parity, years of marriage, and infertility. According to the data presented by the Department of Statistics in Jordan from the Population and Family Health Survey 2017–2018, the desire to have more children strongly relates to the number of living children present. The desire ceases as the number of living children increases. Women above the age of 25 and those with a higher level of education are more likely to use family planning [10]. Similar behavior was demonstrated during the COVID-19 pandemic, as was evident from the results of this study.

Pandemics have a major influence on fertility. The effects will vary with mortality and morbidity and may also vary over different times: short-term versus long-term effects. The psychological and economic burdens that result from epidemics will affect fertility behaviors [11]. Uncertainty about possible risks and mortality will lead to fertility postponement. The possible risk of pregnancy during the pandemic was the main cause for deferring fertility plans, as was evident by the majority of the respondents, which was in line with the recommendations from different fertility societies around the world as well as the recommendations of the Jordanian Society of Fertility and Genetics, which were issued to deal with ART treatments and to prioritize cases during this pandemic, including for cases of oncology patients, older patients, and those with a poor ovarian reserve where time was an important factor regarding their fertility [12] 

Their fears were probably raised from the initial available information that COVID-19 increases morbidity during pregnancy, and thus pregnancy should be avoided. Previous studies have shown that women during pregnancy and puerperium are more vulnerable to infection and are at a higher risk of having severe COVID-19 infection, requiring hospitalization in comparison to non-pregnant or other family members in the same household [9,13,14,15] Vertical transmission of the virus to the baby is possible, but there is no evidence yet that infection during pregnancy has consequences for fetal health [16]. Despite all this evidence, pregnant women have been included in the list of people at moderate risk as a precaution, especially in the third trimester when frequent hospital visits may expose the woman to the infection [17]. Moreover, a higher mortality rate was reported from previous viral outbreaks, such as H1N1 [18,19]. The available information from previous pandemics and the emerging data about the new COVID-19 pandemic have affected the medical bodies’ recommendations and attitudes. This will definitely be reflected in society’s fertility behavior. In addition, several other factors such as infertility, duration of marriage, and age play an important role in fertility preferences and attitudes.

In relation to fertility behavior, our results are similar to other available studies. In a study conducted in Italy, more than 80% of respondents did not plan to have children during the pandemic mainly due to doubts and apprehension about the economic future [20]. Furthermore, in the UK, 71.9% of those who were planning a pregnancy intentionally postponed their plans. Their main concern was about the changes in pregnancy care and about the possible effect of the virus on maternal and fetal health [21]. Regardless of the cause, the percentages were near to our findings, where 73.2% of those who wanted more children were not trying to conceive during the pandemic. Meanwhile, in a study conducted in China, 66.2% of the participants did not change their fertility intentions to have children, but the intentions of 33.8% of them were affected by the pandemic. Most of them were worried about being infected with COVID-19 either during antenatal care or in a public place [22]. The differences in the percentages could be related to cultural issues and local policies. Comparing the fertile and the infertile among those who want more children, 84% of the fertile and 60% of the infertile were not trying to conceive during the pandemic. None of the previous studies explored the differences between these two categories. It is expected that infertile women are less likely to change their decision.

In view of assisted reproductive technique (ART), the participants were asked that, regardless of their fertility status, if ART was needed, would they proceed during the pandemic. Almost all of them answered that they would not, while 97.4% of the fertile but 89.9% of the infertile were not willing to use ART if needed during the pandemic. This goes along with our previous result where the infertile are more likely to continue their plans during the pandemic. This reflects how essential fertility treatment is for infertile women such that, despite the pandemic and the possible associated risks, they are still interested in proceeding with assisted reproductive technique to achieve their desired fertility. The closure of fertility clinics during the COVID-19 pandemic was found to be stressful for fertility patients [23]. This emphasizes our findings that the infertile women are more likely than the fertile to continue their fertility plans during the pandemic. However, what is surprising is that younger women were more likely to try to conceive, while we expected them not to. This is contrary to the findings from previous epidemics where younger women (<25) were more likely than older women to postpone pregnancy [24]. In this situation, education level seems to play a large influence. It is known from previous pandemics that high-quality parents postpone fertility until the pandemic is over [11]. The advice of physicians also has a role in planning and deciding fertility treatment. For example, in a survey conducted in our region for doctors in reproductive medicine (Iraq Fertility Society), 66% of practitioners were happy to resume treatments during the pandemic, although it was seemingly continuing [25].

One limitation of this study was that participants answered the questionnaire during the lockdown, when fertility clinics were closed, which might have affected their answers. Conversely, our study was characterized by a relatively good size of 814 women, where other studies included 500 or less. Furthermore, we addressed the awareness of the risk of COVID-19 infection on pregnancy and fertility behavior in addition to analyzing the differences between fertile and infertile women and the differences between spontaneous conception versus ART. Future behavioral studies might answer questions such as the level of women that perceived their personal risk related to COVID-19 infection, how many tried and failed to get pregnant, or those who got pregnant. Another suggested future study is to assess, retrospectively, the data from fertility clinics and compare them with data before the pandemic, to determine if assisted reproduction outcomes were affected or not. Another recommendation is to raise awareness regarding the published scientific results, to educate society about the facts and myths related to infertility and reproductive assistance and the effects of certain diseases and/or pandemics in fertility.

In preparation for any future pandemic, setting priorities and good preparation in the medical field are essential, particularly in light of the alterations in fertility treatment practices and conception preferences due to the COVID-19 pandemic. This article adds information to the existing literature related to the pandemics’ effects on reproductive concerns and rights and the support strategies and social education that should be available to those seeking help in natural and artificial conception during any possible future pandemic.

## 5. Conclusions

This study concludes that fertility behavior of women in Jordan changed during the pandemic. Their desire to naturally conceive and use ART declined, as they believed that there were potential risks related to conceiving during the pandemic. However, the effects appeared to be greater among the general fertile population rather than for the infertile.

## Figures and Tables

**Table 1:** General characteristics of the participating women (n = 814).

		Number (Percentage)
Age	20–25	94 (11.5%)
	26–30	179 (22%)
	31–35	199 (24.4%)
	36–40	192 (23.6%)
	>40	147 (18.1%)
Education	High school	132 (16.2%)
	Diploma	106 (13%)
	Bachelor’s	455 (55.9%)
	Higher education	121 (14.9%)
J Occupation	Housewife	480 (59%)
	Employee	334 (41%)
Years of marriage	<1	48 (5.9%)
	1–2	52 (6.3%)
	2–3	53 (6.5%)
	3–4	52 (6.4%)
	4–5	42 (5.2%)
	5–6	40 (4.9%)
	>6	528 (64.9%)
Parity	P0	79 (9.7%)
	P1	143 (17.6%)
	P2	195 (24%)
	P3	171 (21%)
	≥P4	226 (27.8%)
Place of residency	City	667 (81.8%)
	Village	120 (14.7%)
	Refugee camp	27 (3.3%)

## Data Availability

Data are available upon request.
